# Left atrial functional impairment as a predictor of atrial fibrillation: insights from cardiac CT

**DOI:** 10.1007/s00330-025-11348-z

**Published:** 2025-01-21

**Authors:** Doron Aronson, Daniel Perlow, Sobhi Abadi, Jonathan Lessick

**Affiliations:** 1 Rappaport Faculty of Medicine, Technion - Israel Institute of Technology, Haifa, 3525422 Israel; 2 Cardiology Department, Rambam Health Care Campus, Haifa, Israel; 3 Medical Imaging Department, Rambam Health Care Campus, Haifa, Israel

**Keywords:** Atrial function, Atrial fibrillation, Atrial remodeling, Computed tomography, Multidetector

## Abstract

**Objectives:**

A strong association exists between left atrial (LA) structural remodeling and the development of atrial fibrillation (AF). The role of LA function in AF prediction remains unclear. We studied the relationship between LA function and incident AF using cardiac CT.

**Materials and methods:**

We retrospectively analyzed patients who underwent multiphasic cardiac CT. LA volumes and parameters of LA global, reservoir and booster function were calculated. The association between measures of LA function and incident AF was analyzed using multivariable Cox regression adjusting for clinical variables, LA volume and left ventricular function.

**Results:**

1025 patients (age 64 years ± 14) were evaluated. Over a median of 3.9 years, 90 patients developed AF. There was a significant association between LA total emptying fraction (adjusted hazard ratio (HR) 1.05; 95% CI: 1.02–1.05 per 1% decrease, *p* < 0.001), LA reservoir function (HR 1.04; 95% CI: 1.02–1.06 per 1 mL/m^2^ decrease in LA expansion index, *p* < 0.001) and passive LA emptying (HR 1.08; 95% CI: 1.03–1.13 per 1% decrease in LA passive emptying fraction, *p* < 0.001) with incident AF, but no association with LA booster function. Incorporating LA function into predictive models improved risk stratification beyond clinical variables and LA volume. Mediation analysis demonstrated that 46% of the effect of LA volume on AF was mediated via LA dysfunction.

**Conclusion:**

LA functional impairment is common even in patients with normal LA volume and provides additional prognostic information for AF risk. The findings underscore the significance of LA mechanical dysfunction in the pathogenesis of AF.

**Key Points:**

***Question*** *A strong association exists between left atrial structural remodeling and incident atrial fibrillation. The role of left atrial function in atrial fibrillation prediction remains unclear.*

***Findings*** *Left atrial reservoir and passive emptying function (but not booster function) predict incident atrial fibrillation independent of left atrial volume and clinical risk factors.*

***Clinical relevance*** *Left atrial functional impairment precedes the development of atrial fibrillation. Measures of left atrial reservoir and passive emptying function are independent predictors of incident atrial fibrillation.*

**Graphical Abstract:**

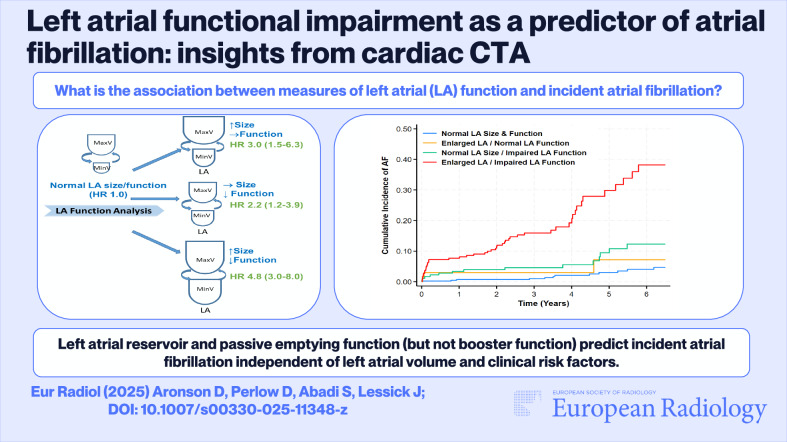

## Introduction

Atrial fibrillation (AF) is the most common cardiac sustained arrhythmia, with an estimated prevalence ranging between 2% and 4%, and is associated with substantial morbidity and mortality [1]. There is a strong association between left atrial (LA) structural remodeling and the development of AF [2, 3]. Maximal LA volume, a simple marker of LA remodeling, has been shown to be incremental to clinical risk factors for the prediction of incident AF [4, 5].

LA remodeling is a complex spectrum of pathophysiological changes that affect not only LA atrial structure but is also linked to functional impairments and altered electrophysiological properties [6–8]. However, few data are available with regard to the role of LA function for AF prediction [9–11]. Thus, it remains unknown whether LA function contributes to the development of AF beyond clinical risk factors and LA enlargement.

Contrast-enhanced cardiac CT allows for isometric 3D geometric measurements which offer rapid, automatic voxel-based calculations of various LA volumetric measurements [12–15] and offer improved sensitivity and accuracy in the evaluation of LA remodeling and function [12, 13]. In the current study, we perform a comprehensive analysis of LA remodeling and function using cardiac CT, with the aim of testing whether LA functional parameters provide incremental information for the prediction of AF over clinical risk factors and LA volume.

## Materials and methods

The study was initiated after receiving approval from the Rambam Health Care Campus Institutional Review Board and Ethics Committee on Human Research (Approval ID: RMB-0057-15). The need for written informed consent was specifically waived by the because of the retrospective nature of the study.

From our database of patients who had undergone cardiac CT between January 2012 and December 2019, we retrospectively identified consecutive patients who had undergone a cardiac CT examination using spiral scanning with retrospective gating, Scanning was performed on a dual-source Somatom Definition Flash scanner (Siemens Healthcare), with a temporal resolution of approximately 70 ms. Data was reconstructed every 5% of the cardiac cycle to provide 20 phases per study. We excluded patients with atrial fibrillation during the study, unstable patients, and insufficient CT quality.

Data were analyzed on a dedicated CT workstation (IntelliSpace Portal, version 11, Philips Healthcare) using the CT Comprehensive Cardiac Analysis software (Philips Healthcare). Retrospective data were analyzed using fully automatic segmentation of the heart chambers, producing phasic volume curves of each heart chamber. LA volume-based indices of LA function were calculated as previously described [15]. The agreement between the automated and manual measurements was high (Supplementary Material).

The primary endpoint of the study was incident AF after the cardiac CT. Incident AF ascertainment was accomplished by a comprehensive review of the medical records of all study participants.

The study participants were divided into three groups: Patients without AF at any time, patients with a history of AF prior to the cardiac CT, and patients without a history of AF but with incident AF after the cardiac CT. We first analyzed the association between measures of LA volume and function with previous history of AF. Then, after excluding patients with a history of AF, we studied the association between measures of LA volume and function with incident AF.

### Indicators of LA remodeling and function

Maximum LA volume was defined as LA volume at end-systole just before mitral valve opening and was indexed to the body surface area to derive the LA volume index (LAVI).

Indexes of global LA function, LA reservoir function, early passive filling and LA booster function are summarized in Supplementary Table 1. Global LA function was assessed by LA total emptying function (LATEF) and LA function index (LAFI), which normalizes function to stroke volume and is rhythm-independent [8, 12, 13, 16–19]. Measures of reservoir function included reservoir volume index (ResVi) and LA expansion index (LAEI), representing the relative LA volume changes during the reservoir phase.

Early passive LA function was characterized by passive LA emptying fraction [15, 20, 21]. LA booster function was characterized by LAEF_Booster_ [20, 22].; Other surrogates of LA booster function included LA booster contribution to LV stroke volume.

Based on previous studies, LA enlargement was defined as LAVI ≥ 62 mL/m^2^ [14], and normal global LA function was defined as LATEF ≥ 40% [13, 19].

### Statistical analysis

Continuous variables are presented as mean ± SD or medians (with interquartile ranges), and categorical variables as numbers and percentages. The relationship between various parameters of LA volume and function were assessed by Pearson correlation.

The associations between LA function and clinical characteristics and CT data were assessed with the use of univariable linear regression. Variables found to have a univariable association with LA function at the *p* ≤ 0.1 level (Wald test) were used in multiple linear regression. Partial correlation coefficients were used to estimate the proportion of variance of dependent variables explained by each independent variable.

Kaplan–Meier plots were used to assess the event-free probability of AF associated with LA volumes and function, and the log-rank test was used to compare the curves. The association between LATEF and the endpoint of incident AF was then analyzed using Cox regression analysis. The following clinical covariables known to predict AF [23, 24] were used in a multivariate stepwise Cox proportional hazards model: age, sex, body mass index, previous heart failure, hypertension, diabetes mellitus, coronary artery disease, multivessel coronary artery disease, previous myocardial infarction, previous coronary artery bypass surgery, left ventricular ejection fraction, and severe aortic or mitral valve disease. In addition, measures of LA size and LA function, dichotomized above or below the upper normal value or used as continuous variables, were considered in the model. To address the risk of model overfitting, we also computed a more parsimonious multivariate Cox model that included fewer variables (Wald test; *p* < 0.05 in the univariate analysis).

The incremental value of LA volume and function over common clinical predictors of AF was assessed by exploring changes in the global chi-square values in sequentially constructed multivariate Cox models. We compared models using the Akaike information criterion, Bayes information criterion (likelihood measures in which lower values indicate better fit and in which a penalty is paid for increasing the number of variables in the model), and Harrell C index. Comparison of C indexes was made as proposed by Newson [25].

Restricted cubic spline transformations of the continuous independent variable were implemented to detect a potential nonlinear relationship of continuous variables, using three knots placed at default locations. Statistical significance of nonlinearity (i.e., curvature) was tested by comparing the cubic spline model with the linear model, and *p*-values of < 0.05 were regarded as statistically significant nonlinear relationship between the exposure and the outcome.

To determine whether LA dysfunction mediates part of the association between LA size and risk of AF, we performed a causal mediation analysis where LA volume was the independent covariable, LA function was the mediator, and incident AF was the dependent variable. Attenuation of the regression coefficient for LA volume in the mediation model compared with the logistic regression model provides qualitative evidence for mediation [26]. We used a 2-way decomposition and estimated the multivariable-adjusted direct and indirect effects (i.e., mediation effect) and percentage (%) mediated by LA function. Multivariable adjusted (for all predictors of AF) direct and indirect effects (i.e., mediation effect) are reported, with calculation of 95% CIs using bootstrapping with 500 resamples. Statistically significant mediation was determined if the indirect effect was significantly different from zero.

Differences were considered statistically significant at the 2-sided *p* < 0.05 level. Statistical analyses were performed using STATA Version 18.0.

## Results

The study cohort comprised 1067 patients in sinus rhythm, undergoing spiral cardiac CT from January 2010 to June 2018. We excluded 42 patients due to non-sinus rhythm or poor CT quality.

**Fig. 1:**
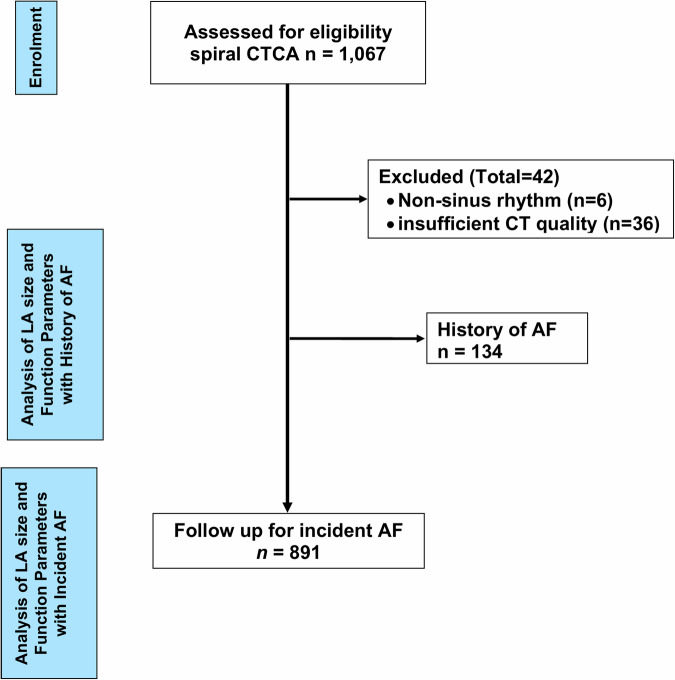
CONSORT flow diagram of the study cohort

Of the remaining 1025 study participants (mean age 64 years ± 14, 60% men) were evaluated. Of these, 801 were without evidence for AF at any timepoint, 134 were with a history of AF prior to the cardiac CT, and 90 were without a history of AF but with incident AF after the cardiac CT (Fig. 1).

The clinical and cardiac CT characteristics of the three study groups are summarized in Table 1. Patients with previous AF or incident AF were older, more likely to be hypertensive and with history of myocardial infarction and heart failure. LA volume was larger and LA functional indexes were impaired in patients with previous or incident AF. LA functional parameters were remarkably similar in patients with previous and incident AF.

**Table 1:** Baseline characteristics and CT measurements according to AF status

Characteristics	Previous AF (*n* = 134)	No AF (*n* = 801)	Incident AF (*n* = 90)	*p*-value
Age (years)	71 ± 12	62 ± 14	70 ± 12	0.002
Male gender	85 (63)	483 (60)	53 (59)	0.74
Hypertension	107 (80)	520 (65)	81 (90)	< 0.001
Diabetes mellitus	51 (38)	280 (35)	36 (40)	0.59
Coronary artery disease	60 (45)	322 (40)	42 (47)	0.4
Multivessel coronary disease	44 (33)	215 (27)	34 (38)	0.047
Previous CABG	38 (28)	181 (23)	25 (28)	0.23
Previous myocardial infarction	40 (30)	206 (26)	35 (39)	0.02
Previous heart failure	33 (25)	89 (11)	24 (27)	< 0.001
**CT measurements**				
LVEDVI (mL/m^2^)	93 ± 29	87 ± 25	95 ± 34	0.002
LVESVI (mL/m^2^)	38 ± 23	36 ± 22	44 ± 32	0.02
LVEF (%)	60 ± 13	60 ± 13	58 ± 14	0.24
LVMI (g/m^2^)	88 ± 26	78 ± 26	91 ± 28	< 0.001
LAESVI (mL/m^2^)	68 ± 18	56 ± 15	68 ± 23	< 0.001
LAEDVI (mL/m^2^)	48 ± 20	34 ± 14	48 ± 19	< 0.001
LAV_pre-A_ volume index (mL/m^2^)	60 ± 18	47 ± 14	59 ± 17	< 0.001
LAEF_Passive_ (%)	12.8 ± 6.1	17.0 ± 7.3	12.5 ± 5.6	< 0.001
LAEF_Contractile_ (%)	20.0 ± 9.5	23.8 ± 8.5	20.0 ± 8.5	< 0.001
LA early passive contribution to LV stroke volume (%)	16.1 ± 6.8	18.9 ± 8.3	16.6 ± 7.7	0.0002
LA booster contribution to LV stroke volume (%)	24.0 ± 11.5	26.0 ± 9.6	24.9 ± 12.7	0.09
LAEF_Total_ (%)	32.3 ± 12.5	40.2 ± 11.2	31.6 ± 11.6	< 0.001
LA expansion index (mL/m^2^)	50 ± 30	73 ± 30	51 ± 26	< 0.001
LAFI	54 ± 33	79 ± 34	51 ± 28	< 0.001

Values are presented as *n* (%), mean ± SD or median (interquartile range)

*LVEDVI* left ventricle end-diastolic volume index, *LVESVI* left ventricle end-systolic volume index, *LVEF* left ventricle ejection fraction, *LVMI* left ventricle mass index, *LAESVI* left atrial end-systolic volume index, *LAEDVI* left atrial end-diastolic volume index, *LAV* left atrial volume, *LAEF* left atrial emptying fraction, *LAFI* left atrial functional index

### Relationship between LA remodeling and LA function

LA enlargement was present in 36% of patients. There was a significant but moderate inverse relationship between LAVI and global LA function, measured by LATEF (Pearson correlation coefficient: −0.57; Supplementary Fig. 1A). This relationship was also moderate for LAEF_passive_ (Pearson correlation coefficient: −0.50) but weak for LAEF_active_ (Pearson correlation coefficient: −0.30) and reservoir volume index (Pearson correlation coefficient: 0.17). Among subjects with normal LAVI, 20% had impaired LATEF, while 8% of those with increased LAVI had normal global LA function. Table 2 displays the clinical and CT predictors of LATEF. Independent predictors of LA dysfunction included age, male gender, history of AF, valvular heart disease, reduced ejection fraction, increased LV mass index, and increased LAVI. The variables in the model accounted for approximately half of the variance in LATEF (*R*^2^ = 0.49).

**Table 2:** Multivariable linear regression analysis for LA total emptying fraction*

	Univariable		Multivariable	
Baseline parameters	Regression coefficient (SE)	*p*-value	Regression coefficient (SE)	*p*-value
Age (per 10 years)	−2.81 (0.24)	< 0.001	−1.25 (0.20)	< 0.001
Male gender	2.38 (0.76)	< 0.001	2.03 (0.64)	0.001
Hypertension	−4.69 (0.79)	< 0.001	–	–
Diabetes mellitus	−2.36 (0.77)	0.002	–	–
Valvular heart disease	−8.81 (0.86)	< 0.001	−2.66 (0.76)	0.001
LV ejection fraction (per 1% increase)	1.51 (0.16)	< 0.001	0.77 (0.14)	< 0.001
LV ejection fraction squared (per 1% increase)	−0.01 (0.001)	< 0.001	−0.005 (0.001)	< 0.001
LV mass index (per 1 g/m^2^ increase)	−0.16 (0.01)	< 0.001	−0.03 (0.01)	0.03
LA volume index (per 1 mL/m^2^ increase)	−0.42 (0.02)	< 0.001	−0.27 (0.02)	< 0.001
History of atrial fibrillation	−7.06 (1.11)	< 0.001	−2.59 (0.85)	0.003

* The model explains 49% of the variability in LATEF

Supplementary Fig. 1B shows that the two main LA functions, early filling and contractile, poorly correlate (Pearson *r* = 0.02), indicating that these components of LA function may not fail in parallel.

### Relationship between LA function and previous AF

We first examined the association between LA size and function and history of AF using a multivariable logistic regression model (Table 3). Both increased LA size with normal LA function and impaired LA function with normal LA size were associated with a history of AF. LATEF was also associated with history of AF when used as a continuous variable.

**Table 3:** Logistic regression model for the association of LA size and function with previous diagnosis of AF*

	Univariable		Multivariable	
LA size and function parameters	OR (95% CI)	*p-*value	OR (95% CI)	*p*-value
Binary definitions				
Normal LAESVI, normal LATEF	1.0	–	1.0	–
Increased LAESVI, normal LATEF	3.01 (1.45–6.29)	0.003	2.74 (1.28–5.84)	0.009
Normal LAESVI, reduced LATEF,	2.18 (1.20–3.94)	0.01	2.09 (1.12–3.88)	0.02
Increased LAESVI, reduced LATEF	4.8 (2.99–7.96)	< 0.001	4.26 (2.45–7.41)	< 0.001
Continuous definitions				
LAESVI (per 1 mL/m^2^ increase)	1.03 (1.01–1.04)	< 0.001	1.03 (1.01–1.04)	0.001
LATEF (per 1% increase)	0.97 (0.96–0.99)	0.009	0.97 (0.95–0.99)	0.006

* Adjusted for age, sex, body mass index, hypertension, diabetes mellitus, coronary artery diseases, coronary artery bypass surgery, left ventricular ejection fraction, valvular heart disease, and left atrial volume index

*LAESVI* left atrial end-systolic volume index, *LATEF* left atrial total emptying fraction

### Relationship between LA function and incident AF

The relationship between LA size and function and incident AF was examined in the subset of patients without previous AF (*n* = 891). Over a median follow-up of 3.9 years (IQR 3.8–4.1) AF occurred in 90 patients (10.1%).

Based on the concordance of the volumetric and the functional properties of the LA, 423 patients (47%) had normal LA volume and function, 69 patients (8%) had dilated LA with normal function, 178 patients (20%) showed normal LA volume and impaired LA function, and 221 patients (25%) had both a dilated LA and impaired LA function.

**Fig. 2:**
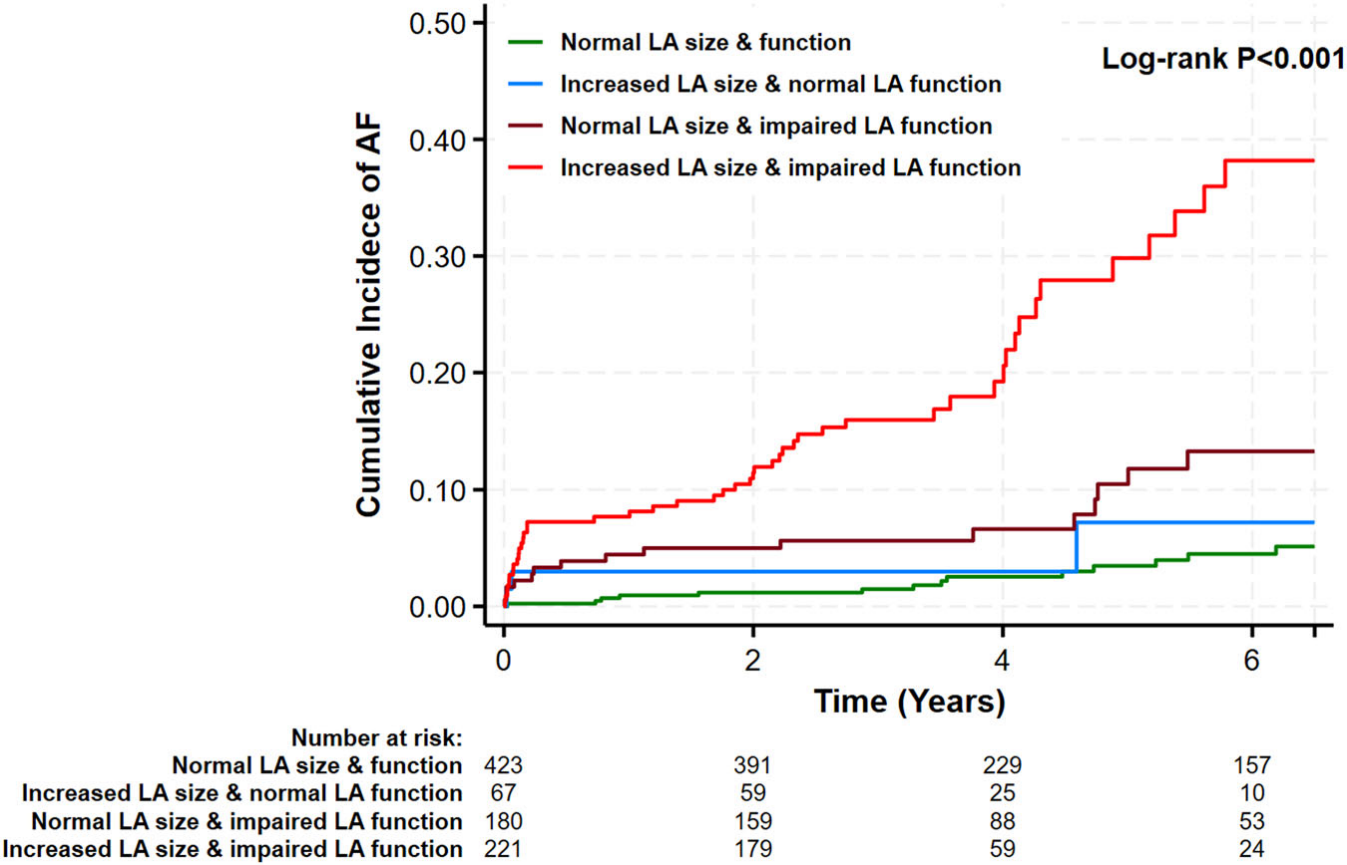
Kaplan–Meier estimates of incident atrial fibrillation according to left atrial size and left atrial function

The Kaplan–Meier estimates of incident AF events at 6 years (Fig. 2) were low in patients with normal LA size and function (5.1% [95% CI: 3.0–8.8%]), modestly increasing to 9.9% (95% CI: 3.7–24. 8%) in patients with enlarged LA volume and normal LA function and 12.3% (95% CI: 7.1–20.9%) in patients normal LA size and impaired LA function. Incident AF events markedly increased in patients with both increased LA volume and impaired LA function 38.2% (95% CI: 28.2–50.3%) (Fig. 2).

**Fig. 3:**
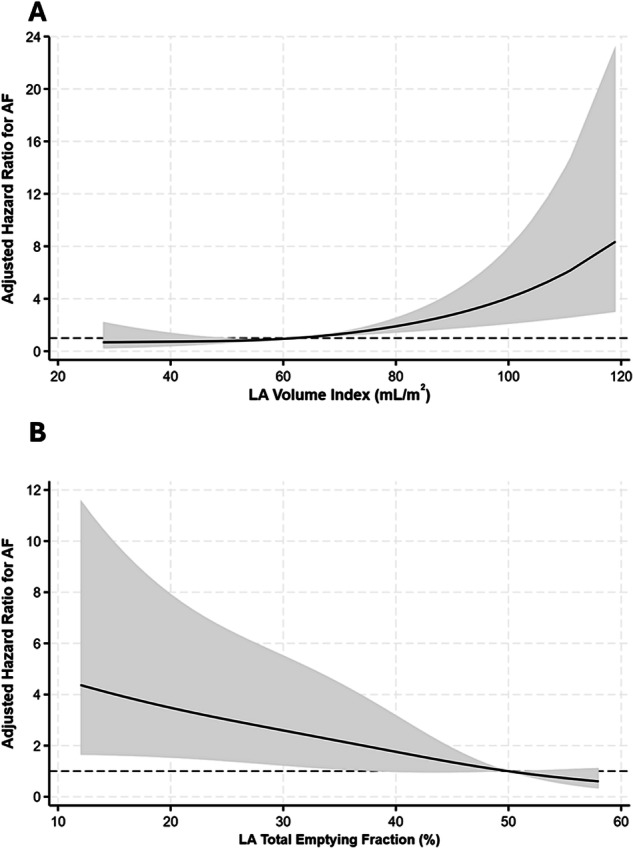
Spline function graph of the relationship between incident atrial fibrillation and (**A**) left atrial end-systolic volume index (P-nonlinearity = 0.82) and (**B**) left atrial total emptying fraction (P-nonlinearity = 0.34)

Spline regression analysis demonstrated a near linear association between both LAVI and LATEF with the hazard of AF (Fig. 3). Therefore, we also explored the relationship between measures of LA function as continuous variables and incident AF.

Table 4 shows the results of Cox proportional hazards models for incident AF using various measures of LA function. Overall, measures of global LA function, LA reservoir function and early filling were more predictive of incident AF than measures of LA booster function. After adjustments for clinical variables and LA volume, all measures of LA global function (LATEF, HR 1.05, 95% CI: 1.02–1.05 per 1% decrease), LA early emptying (LAEF_passive_, HR 1.08, 95% CI: 1.03–1.13 per 1% decrease) and reservoir function (LA expansion index, HR 1.04, 95% CI: 1.02–1.06 per 1 mL/m^2^ decrease) were independently associated with incident AF. By contrast, none of the measures of LA booster function was associated with incident AF (LAEF_booster_, HR 1.03; 95% CI: 0.99–1.06 per 1% decrease).

**Table 4:** Cox proportional hazards models for the association of LA size and function with incident AF

	Univariable		Multivariable	
LA functional parameters	HR (95% CI)	*p-*value	HR (95% CI)	*p*-value
Early passive emptying				
LAEF passive (per 1% decrease)	1.13 (1.09–1.18)	< 0.001	1.08 (1.03–1.13)	0.001
LA early passive contribution to LV stroke volume (per 1% decrease)	1.06 (1.03–1.09)	< 0.001	1.04 (1.01–1.07)	0.02
Reservoir function				
Reservoir volume index (per 1 mL/m^2^ decrease)	1.05 (1.02–1.09)	0.002	1.04 (1.01–1.07)	0.02
LA expansion index (per 1 mL/m^2^ decrease)	1.05 (1.04–1.07)	< 0.001	1.04 (1.02–1.06)	< 0.001
LA pre-A volume index (per 1 mL/m^2^ increase)	1.06 (1.04–1.07)	< 0.001	1.08 (1.02–1.15)	0.006
Booster function				
LAEF booster (per 1% decrease)	1.06 (1.03–1.09)	< 0.001	1.03 (0.99–1.06)	0.14
LA booster contribution to LV stroke volume (per 1% decrease)	1.01 (0.99–1.03)	0.44	1.01 (0.98–1.03)	0.52
Global function				
LATEF (per 1% decrease)	1.07 (1.05–1.09)	< 0.001	1.05 (1.02–1.05)	< 0.001
Left atrial function index (per 1 unit decrease)	1.03 (1.02–1.04)	< 0.001	1.02 (1.01–1.03)	< 0.001

The clinical variable-based model had a good discrimination with an AUC of 0.75 (95% CI: 0.71–0.80). A model based only on LA size and function gave similar results. Both LAVI and LATEF improved model fit and increased the C-statistics when added to clinical variables model. The best model included the clinical variables with LA volume and LA function (Table 5). Similar results were obtained with a model adjusting for a smaller set of variables to mitigate the risk of overfitting (Supplementary Table 2).

**Table 5:** Incremental prognostic value analysis of left atrial function over clinical variables and left atrial volume

Model	AIC	BIC	Harrell C index (95% CI)
Clinical variables	1012	1026	0.75 (0.71–0.80)
LAESVI + LATEF	1012	1026	0.77 (0.71–0.82)
Clinical variables + LAESVI	932	990	0.81 (0.76–0.85)*
Clinical variables + LATEF	944	997	0.80 (0.76–0.85)†
Clinical variables + LAESVI + LATEF	928	986	0.82 (0.79–0.87)‡

*AIC* Akaike information criterion, *BIC* Bayes information criterion

* *p* = 0.002 compared with the model containing only the clinical variables

† *p* = 0.006 compared with the model containing only the clinical variables

‡ *p* < 0.001 compared with the model containing only the clinical variables

#### Mediation analysis

**Fig. 4:**
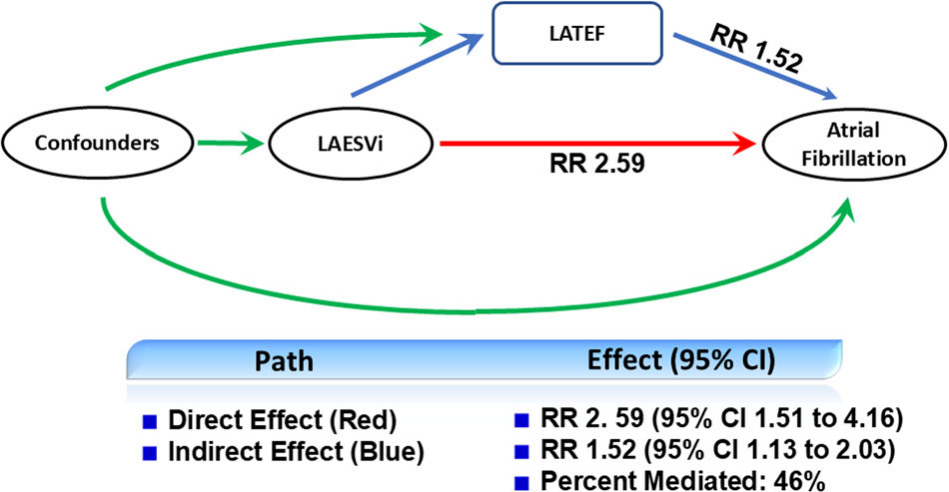
Directed acyclic graph illustrating possible structural relationships between LA volume and incident AF. Confounders denotes common causes (confounding factors). The mediated effect is represented by the pathway from LA volume to incident AF that goes through LA function (blue arrows). The direct effect (red arrow) is the pathway from LA volume straight to incident AF. RR, risk ratio

In a causal mediation analysis (Fig. 4), LA function was significantly associated with incident AF after adjustment for the other risk variables (*p* < 0.001) and was a mediator of the association between LA volume and incident AF (46% of the total effect of LA volume).

## Discussion

The present study reports a comprehensive evaluation of the association between LA remodeling and functional impairment with the risk of AF. The principal findings of this study are: (1) LA volume and function are similar between patients with history of paroxysmal AF and those who subsequently developed AF; (2) There is only a moderate association between LA volume and function; (3) Measures of LA reservoir and passive emptying correlate more strongly with incident AF than measures of LA booster function; (4) Left atrial function adds prognostic information to clinical risk factors and LA volume. (5) Nearly half of the effect of LA volume on AF risk is mediated by the associated LA dysfunction. From the pathophysiological perspective, the study highlights the importance of LA mechanical dysfunction (irrespective of LA remodeling) in the pathogenesis of AF.

### Both LA remodeling and dysfunction contribute to AF risk

LA remodeling occurs in response to a chronic pathological stimulus such as hypertension and increased fat mass and is associated with an increased risk of AF events [2, 6]. LA enlargement is often used clinically as a simple measure of LA structural remodeling. However, LA structural remodeling is the result of a complex pathophysiological process that involves alterations in cardiomyocyte, fibroblast, and non-collagen infiltrative compartments of the atrium [7, 27] and is accompanied by functional LA impairment.

The development of AF can contribute to adverse atrial remodeling and dilatation [28]. However, this phenomenon has been demonstrated to occur predominantly in persistent AF [29]. We observed a remarkable similarity in LA size and function between individuals with previous paroxysmal AF episodes and those at risk for future AF. These results suggest that in patients with a low AF burden, AF is a consequence of a dysfunctional LA rather than its cause. In these patients, LA enlargement is secondary to associated processes causing LA pressure and/or volume overload [30, 31].

There is growing evidence that a large proportion of patients who develop AF (particularly paroxysmal AF) do not have an enlarged LA [32], suggesting that LA dysfunction per se (without overt LA remodeling) increases the propensity to develop AF. We identified global LA dysfunction in approximately half of subjects with normal LAVI. Furthermore, the increased LA volume explained only ~14% of the variability in LATEF, the remainder determined by other risk factors such as age, LV systolic function, LV mass and valvular heart disease.

We observed a linear association between both LATEF and LA reservoir function with incident AF, indicating a close relationship between LA mechanical properties and the risk of incident AF. Finally, nearly 50% of the effect of LA volume on the risk for AF was mediated via LA dysfunction. Thus, although LA enlargement and LA dysfunction share common risk factors, they do not necessarily develop in parallel, and subclinical LA myopathy is a risk factor for incident AF.

### AF risk and components of LA functional impairment

In animal models [33] and limited human studies [34–36], failure of passive emptying precedes that of contractile function. In early-stage LA failure, a reduction in early passive filling is often accompanied by a compensatory augmentation of booster-pump function that is driven by an increased LA volume at the end of diastasis that stimulates the Frank-Starling mechanism [15]. At this stage, global LA function may be preserved. Over time, atrial myocyte loss and atrial fibrosis progressively diminish booster-pump function, and global LA function becomes impaired [8, 15].

The current study demonstrates, however, that impairment of reservoir function or passive emptying is sufficient to increase AF risk independent of clinical variables and LA volume. The fact that reservoir function and early passive filling represent earlier manifestations of LA disease [8, 15] explains the stronger association with AF. The lack of association between LA booster function with incident AF probably reflects the fact that LA booster function failure tends to occur later in the course of LA disease. With mild LA disease, the reduction in early passive filling results in an increased LA volume at the end of diastasis, and the booster function increases via the Frank-Starling mechanism. At more advanced stages of LA disease, atrial myocyte loss and atrial fibrosis lead to a progressive decline in booster-pump function [8, 15].

LA dilation and myocardial fibrosis causing LA dysfunction and electromechanical conduction delay characterize LA remodeling and form the substrate for AF [37]. However, although assessment of atrial function is becoming increasingly common, currently, risk stratification for the occurrence of AF is based on clinical scores that do not include LA remodeling and function in their algorithms [38–40]. Incorporating LA functional parameters into risk models may further refine the risk and better identify patients at risk for incident AF.

#### Study limitations

It is important to consider several limitations pertinent to the current study. First, this was a single-center post-hoc analysis of our CT data with a relatively short follow-up, and thus, the results must be regarded as hypothesis-generating and exploratory and require validation in other studies. LA volumetric measurements were done using a single vendor software. We were able to detect only symptomatic AF episodes. Some AF episodes may have been missed. However, we would not expect this to lead to a systematic bias in the relationship between measures of LA function and AF.

A further vulnerability is the potential for overfitting in the context of having a small number of events (< 10) per considered variable in the Cox models. Sampling bias may have occurred because patients at higher risk were more likely to be referred for cardiac CT. In addition, there is a bias generated by the study inclusion criteria requiring spiral CT studies.

## Conclusion

LA functional impairment occurs in a large proportion of patients with normal LA volume. Functional measures of the LA, including reservoir function, passive filling and LATEF provide incremental information with regard to the risk of incident AF independent of clinical risk factors and LA volume. From the pathophysiological perspective, the study highlights the importance of LA mechanical dysfunction that accompanies LA remodeling in the pathogenesis of AF.

## Supplementary information

ELECTRONIC SUPPLEMENTARY MATERIAL

## Abbreviations

AF: Atrial fibrillation
LA: Left atrium
LAESVI: Left atrial end-systolic volume index
LATEF: LA total emptying function
LAVI: LA volume index
