# Supplementary material for: Left atrial functional impairment as a predictor of atrial fibrillation: insights from cardiac CT
Source: Eur Radiol. 2025 Jan 21;35(7):3907–16. doi: 10.1007/s00330-025-11348-z (PMC12166001; doi:10.1007/s00330-025-11348-z)

Left Atrial Functional Impairment as a Predictor of Atrial Fibrillation: Insights from Cardiac CT

ELECTRONIC SUPPLEMENTARY MATERIAL

Supplemental Table 1: Volumetric Indexes of Left Atrial Function

Phasic LA Function	Parameter	Calculation
Global function	LATEF (%)<br>LA function index (LAFI)	(LAVmax-LAVmin)/ LAVmax<br>LATEF * stroke volume / LAVI
Reservoir function	Reservoir volume index (ResVi)<br>LA expansion index (LAEI)	(LAESVi – LAEDVi)<br>(LAVmax - LAVmin)/LAVmin)
Early passive filling	$LAEF_{Passive}$	$(LAV_{max}$-$LAV_{pre-a})/LAV_{max}$
Booster pump	$LAEF_{Booster}$<br>LA booster contribution to SV	$(LAV_{pre-a}$ -$LAV_{min})$ /LAV_{pre-a}<br>Atrial kick volume/SV

LA = Left atrium; LAEF = Left atrial emptying fraction; LAEDVi = Left atrial end diastolic volume index; LAESVi = Left atrial end-systolic volume index; LATEF = Left atrial total emptying fraction; LAV = Left atrial volume; SV = Stroke volume

Supplemental Table 2: Cox proportional hazards models for the association of LA size and function with incident AF*

	Univariable		Multivariable*	
**LA functional parameters**	**HR (95% CI)**	***P* value**	**HR (95% CI)**	***P* value**
**Early passive emptying**				
LAEF passive (per 1% decrease)	1.13 (1.09–1.18)	<0.001	1.07 (1.02–1.12)	0.002
LA early passive contribution to LV stroke volume (per 1% decrease)	1.06 (1.03–1.09)	<0.001	1.04 (1.00–1.07)	0.025
**Reservoir function**				
Reservoir volume index (per 1 mL/m^2 decrease)	1.05 (1.02–1.09)	0.002	1.03 (1.00–1.07)	0.038
LA expansion index (per 1 mL/m^2 decrease)	1.05 (1.04–1.07)	<0.001	1.03 (1.01–1.05)	0.002
LA pre-A volume index (per 1 mL/m^2 increase)	1.06 (1.04–1.07)	<0.001	1.07 (1.01-1.13)	0.023
**Booster function**				
LAEF booster (per % decrease)	1.06 (1.03–1.09)	<0.001	1.02 (0.99–1.05)	0.22
LA booster contribution to LV stroke volume (per 1% decrease)	1.01 (0.99–1.03)	0.44	1.01 (0.98–1.03)	0.57
**Global function**				
LATEF (per 1% decrease)	1.07 (1.05–1.09)	<0.001	1.04 (1.02–1.06)	0.001
Left atrial function index (per 1 unit decrease)	1.03 (1.02–1.04)	<0.001	1.01 (1.00–1.02)	0.042

* This model is more parsimonious than in the main text and adjusts for the following variables: age, previous heart failure, hypertension, coronary artery disease, previous myocardial infarction, and severe aortic or mitral valve disease and LA volume index.

Reproducibility of LA volumes and conduit volume

A Bland Altman analysis of automatic and manual measurements of LA volumes indicated a bias of 1.3 mL (95% limits of agreement -2.3 to 4.9 mL) for LAEDV and 1.6 mL (95% limits of agreement -1.2 to 4.4 mL) for LAESV. Measurements of left atrial total emptying fraction revealed a mean bias of -0.016% with 95% limits of agreement -0.13 to 0.10 % (Supplemental Figure 2).

Supplemental Figure 1: (A) Association between left atrial emptying fraction and left atrial volume index and (B) Association between left atrial passive emptying fraction and left atrial booster function.

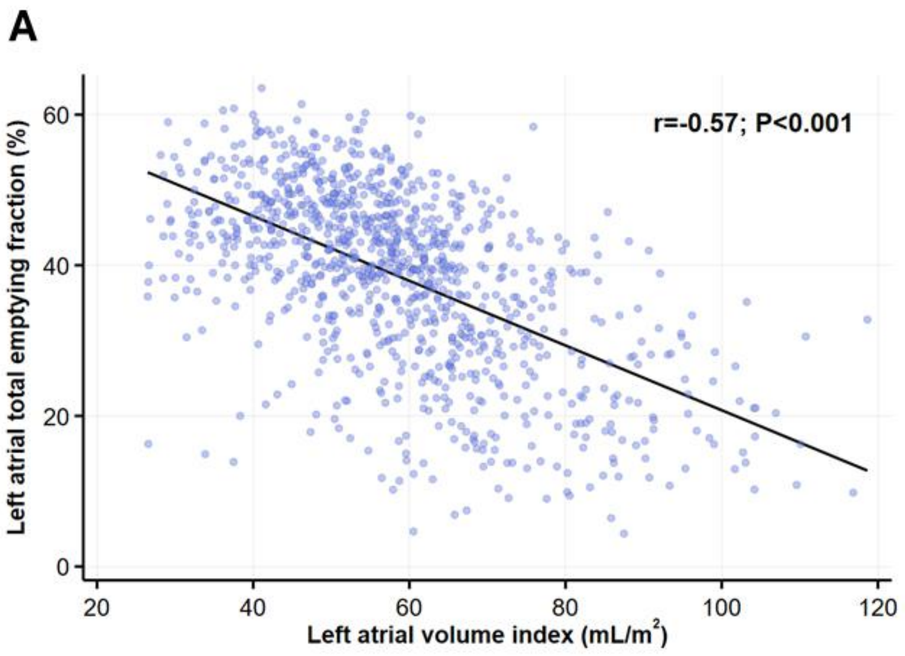

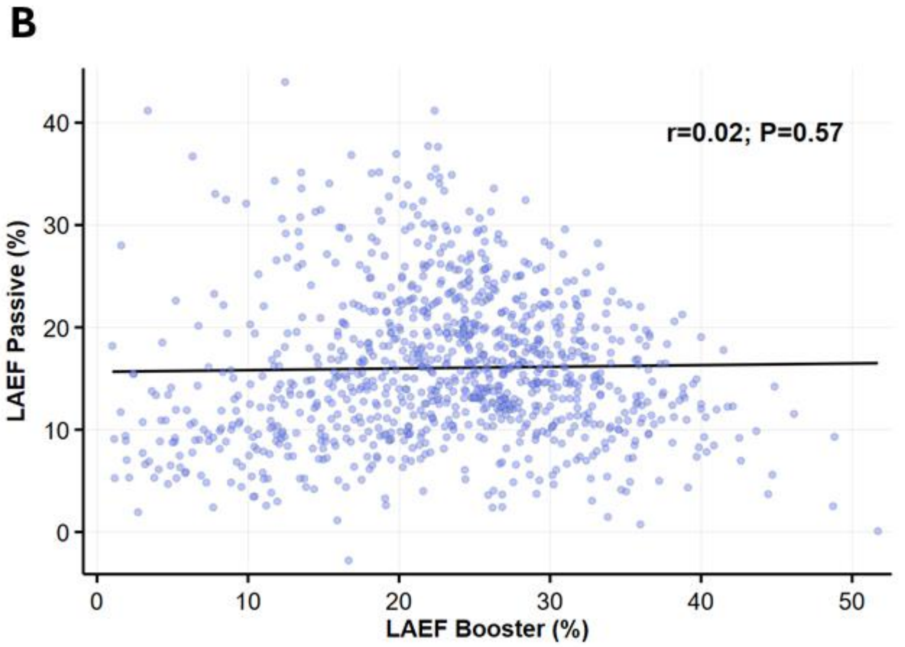

Supplemental Figure 2: Bland-Altman paired difference plots of automatic and manual left atrial total emptying fraction (LATEF) volume volumetric measurements. Dashed gray lines represent limits of agreement (mean ± 1.96 SD) and orange line the bias.

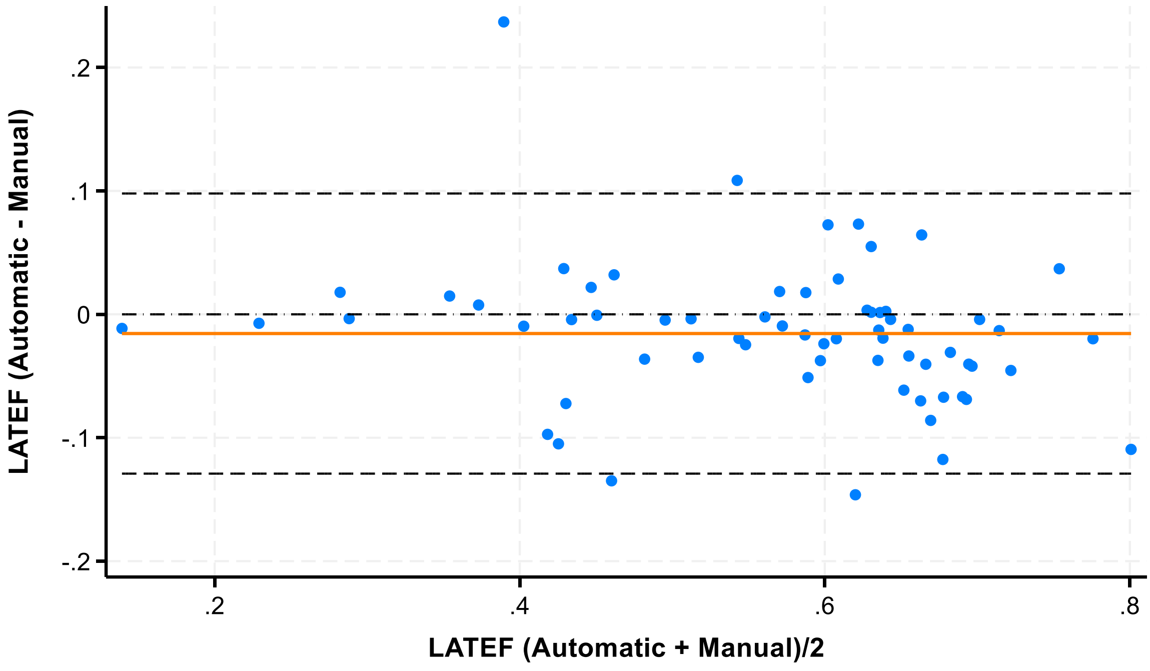

Supplement: Supplementary file 1 — ELECTRONIC SUPPLEMENTARY MATERIAL [file 330_2025_11348_MOESM1_ESM.pdf]
